# The Association Between Repeated Lip Augmentation With Hyaluronic Acid Filler and Recurrence of Herpes Labialis: A Longitudinal Self-controlled Study

**DOI:** 10.1093/asjof/ojag060

**Published:** 2026-04-01

**Authors:** Andrea Felice Armenti

## Abstract

**Background:**

Recurrent herpes labialis (RHL) is a common condition characterized by episodic viral reactivation triggered by local or systemic factors. Hyaluronic acid (HA) lip augmentation is widely performed for aesthetic purposes, yet its potential association with changes in herpes labialis recurrence has not been systematically evaluated.

**Objectives:**

The aim of this study was to investigate whether repeated lip augmentation with HA filler is associated with changes in the incidence of RHL episodes over time.

**Methods:**

A self-controlled longitudinal observational study was conducted in adult patients with a documented history of RHL undergoing lip augmentation with HA filler between 2016 and 2025. Each participant served as their own control, with recurrence rates compared across predefined baseline and posttreatment intervals. The primary outcome was the incidence of clinically typical herpes labialis episodes, expressed as episodes per person-year. Recurrent events were analyzed using generalized estimating equations with a negative binomial distribution.

**Results:**

Ninety patients were included in the final analysis. Baseline incidence was 5.22 episodes per person-year. No significant change in recurrence rate was observed after the first filler session. A statistically significant reduction emerged after the second session (incidence rate ratio [IRR] 0.81; 95% CI, 0.72-0.91; *P* < .001) and became more pronounced after subsequent sessions. After 4 sessions, the IRR was 0.55 (95% CI, 0.45-0.66; *P* < .001). Recurrence rates stabilized after multiple treatments.

**Conclusions:**

In this longitudinal self-controlled cohort, repeated lip augmentation with HA filler was associated with a progressive reduction in RHL incidence after multiple sessions. The clinical relevance of this observed reduction was not predefined. Although causality cannot be established, the temporal pattern and statistical robustness of the findings warrant further investigation.

**Level of Evidence: 4 (Therapeutic):**

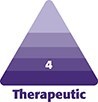

Recurrent herpes labialis (RHL) is a highly prevalent condition caused by latent infection with herpes simplex virus type 1 (HSV-1). Following primary infection, HSV-1 establishes lifelong latency within the trigeminal ganglion, with periodic reactivation leading to clinically evident vesicular lesions of the lip and perioral region.^1^ Despite its benign course in immunocompetent individuals, RHL represents a significant clinical burden because of its frequency, associated discomfort, psychosocial impact, and aesthetic implications.^1,2^ Reactivation of HSV-1 is known to be multifactorial. Established triggers include febrile illnesses, psychological stress, ultraviolet radiation, local trauma, hormonal fluctuations, and immunological perturbations, with individual variability reported in clinical case descriptions.^2-4^ Current management strategies primarily rely on episodic or suppressive antiviral therapies, which reduce viral replication but do not eliminate latency or fully prevent recurrences, and are not routinely adopted long-term in otherwise healthy individuals because of adherence, cost, and risk-benefit considerations.^5^ As a result, there is ongoing interest in identifying additional factors that may influence recurrence patterns, particularly those acting at the local tissue level. However, existing literature has largely focused on pharmacological and immunological interventions, with limited attention given in the literature to structural or biomechanical modifications of the perioral tissues and their potential role in modulating HSV-1 reactivation.^6^

Lip augmentation with hyaluronic acid (HA) filler is among the most commonly performed aesthetic procedures. Although the procedure is generally considered safe, herpes labialis reactivation is discussed in the literature as a potential complication of perioral injections, often prompting prophylactic antiviral use in high-risk patients.^7,8^ To date, the possibility that repeated HA filler treatments might influence the long-term recurrence pattern of herpes labialis has not been systematically investigated. The present study originated not from a prespecified hypothesis but from a recurrent clinical observation. Approximately 1 year after initiating routine lip augmentation treatments in patients with a known history of RHL, multiple patients began to report, spontaneously and independently, a perceived reduction in the frequency of herpes episodes over time. These reports were not solicited nor were patients informed of any expected effect on herpes recurrence.

This observation raised the question of whether repeated lip augmentation with HA filler might be associated with a measurable change in the incidence of RHL episodes. Given the elective nature of aesthetic procedures and the practical challenges of establishing an untreated control group, a longitudinal self-controlled analytical approach was deemed appropriate to explore this association.^9^ Accordingly, the aim of the present study was to quantitatively assess changes in herpes labialis recurrence rates across successive HA lip filler sessions, using each patient as their own control and applying statistical models specifically designed for recurrent events. By transforming a consistent clinical signal into a structured longitudinal analysis, this study contributes preliminary evidence to an underexplored area at the intersection of aesthetic medicine and viral disease recurrence.

## METHODS

### Study Design

This study was designed as a longitudinal self-controlled observational cohort study evaluating the association between repeated lip augmentation with HA filler and the incidence of RHL. The hypothesis emerged from spontaneous patient reports ∼1 year after initiation of lip augmentation treatments. Systematic data collection began thereafter, and baseline herpes labialis frequency was reconstructed retrospectively for the 13 months preceding the first filler session using structured patient interviews and available clinical documentation. Given the absence of a parallel untreated control group, each participant served as their own control, with comparisons performed across predefined temporal intervals relative to treatment exposure. The analytical framework was specifically chosen to address recurrent events, unequal follow-up duration, and within-patient correlation. Data were collected using a standardized checklist for eligibility assessment, baseline characterization, treatment exposure, episode recording, and time-varying confounders (Appendix). Patients were identified from a single-center clinical cohort treated between January 2016 and December 2025.

### Inclusion Criteria

Eligible participants were adults (≥18 years) with a documented history of RHL, defined as 3 to 8 clinically typical episodes per year prior to the first filler treatment, who underwent lip augmentation with HA filler during the study period. Participants were required to have baseline recurrence data available for at least 13 months prior to the first treatment session and to have received no systemic antiviral prophylaxis before or during follow-up.

### Exclusion Criteria

Patients were excluded if they received systemic antiviral therapy at any time during follow-up, with a history of previous lip augmentation before the start of the observation period, required systemic antibiotics for intercurrent infections, underwent surgical procedures (minor or major) requiring perioperative medication, developed acute or chronic systemic illnesses potentially affecting immune function, or had incomplete follow-up data. Patients with fewer than 3 herpes labialis episodes per year were excluded to minimize floor effects and reduce spontaneous variability in recurrence frequency.

Patients meeting any exclusion criterion during follow-up were censored at the time of exclusion and not included in subsequent analyses. A flow diagram summarizing patient identification, exclusions, censoring, and follow-up is provided in Supplemental Figure 1.

### Treatment Protocol

All patients underwent lip augmentation using cross-linked HA filler administered with a standardized vertical injection technique. All treatments were performed using a commercially available HA filler (Stylage Special Lips, Vivacy, Paris, France), delivered with a needle-based vertical injection technique. Injections were placed within the submucosal plane of the lip vermilion. Topical anesthesia was applied prior to injection according to routine clinical practice. Patients were treated in a supine position, and standard postprocedural care recommendations were provided, including avoidance of local trauma and manipulation in the immediate posttreatment period. Injection volumes followed a consistent anatomical distribution per session, with minor variability allowed according to individual anatomy: ∼0.2 ± 0.1 mL was injected per right hemilip of the upper lip, 0.2 ± 0.1 mL per left hemilip of the upper lip, and 0.2 ± 0.1 mL in the lower lip, per session.

The treatment schedule included a first session at baseline, a second session ∼6 months later, and subsequent sessions at ∼12-month intervals. Up to 7 sessions were included in the longitudinal analysis when available.

### Outcome Definition

The primary outcome was the incidence of herpes labialis episodes, defined as clinically typical vesicular or erosive lesions of the lip or perioral area, preceded or accompanied by prodromal symptoms (eg, burning or tingling) and lasting ≥48 h. The outcome was restricted to clinically typical herpes labialis involving the lip vermilion; perioral eruptions outside the vermilion border were not systematically captured and were therefore not included in the analysis. Virological confirmation was not routinely performed, consistent with real-world management of RHL in patients with established disease history. Episodes were identified through standardized patient interviews and clinical records. Use of topical acyclovir during episodes was permitted and recorded; no patient received systemic antiviral therapy.

### Baseline and Follow-Up Periods

Baseline recurrence frequency was assessed over the 13 months preceding the first filler session. The baseline period was defined as the 13 months preceding the first filler session to ensure temporal proximity to follow-up while capturing seasonal variability in herpes labialis recurrence. Longer retrospective baselines were not consistently available or reliably documented across patients and were therefore not used. Follow-up time was partitioned into predefined intervals corresponding to treatment exposure. These included a baseline interval covering the 13 months preceding the first treatment, followed by post-first session (0-6 months), post-second session (6-18 months), post-third session (18-30 months), post-fourth session (30-42 months), and post-fifth or subsequent sessions (≥42 months). For each interval, the number of herpes labialis episodes and the corresponding person-time at risk were calculated.

### Statistical Analysis

Patient characteristics were summarized using means and standard deviations for continuous variables and frequencies with percentages for categorical variables. Incidence rates were expressed as episodes per person-year.

### Primary Analytical Model

To model RHL episodes while accounting for within-patient correlation and variable follow-up duration, generalized estimating equations (GEE) with a negative binomial distribution and log link function were employed.^10,11^ The expected number of episodes for subject *i* in interval *j* was modeled using GEEs with a negative binomial distribution, accounting for within-patient correlation and variable follow-up duration; full model specification is provided in Supplemental Figure 2.

An exchangeable working correlation structure was specified to account for repeated observations within individuals. Results were reported as incidence rate ratios (IRRs) with 95% CI. Baseline served as the reference category.

### Trend and Plateau Analysis

To assess whether recurrence rates changed progressively with increasing treatment exposure, a trend analysis was performed by modeling treatment exposure as an ordinal variable (session step). Additionally, a piecewise regression model was fitted to test for a potential plateau effect after repeated treatments. This model included a linear term for session number and an additional term allowing slope modification beyond the fifth session. Statistical significance of the post-fifth session term was interpreted as evidence of attenuation or stabilization of the treatment-associated effect.

### Sensitivity Considerations

Analyses were designed to be robust to heterogeneity in individual response patterns, variable follow-up duration and seasonal variability in herpes labialis recurrence. No imputation was performed for missing outcome data and analyses were conducted on available person-time.

### Software

All statistical analyses were conducted using IBM SPSS Statistics (version 29.0; IBM Corp., Armonk, NY) for data management and descriptive analyses, and R (version 4.3.1; R Foundation for Statistical Computing, Vienna, Austria) with the *geepack* and *stats* packages for GEE modeling. All tests were 2-sided, and a *P*-value <.05 was considered statistically significant.

### Ethical Considerations

All data were analyzed in anonymized form. The study was conducted in accordance with the Declaration of Helsinki and applicable data protection regulations. Given the observational nature of the study and the use of routinely collected clinical data, formal ethics committee approval was not required. All participants provided informed consent for the use of their clinical data for research purposes prior to inclusion in the study. Key elements of the study design, data structure, exposure timing, outcome definition, and statistical analysis are summarized in Table 1 to facilitate reproducibility and methodological transparency.

**Table 1.** Overview of Study Design, Population, and Statistical Analysis

Domain	Description
Study design	Longitudinal self-controlled observational cohort study; each participant served as their own control
Study setting	Single-center cohort, 2016-2025
Study population	Adults ≥18 years with 3-8 herpes labialis episodes/year
Sample size	141 enrolled; 90 included in final analysis
Sex distribution	92.2% female (83/90)-7.8% male (7/90)
Age at baseline	Mean 38.1 ± 9.8 years (range, 21-55 years)
Exposure	Repeated hyaluronic acid lip filler (vertical technique), up to 7 sessions
Treatment schedule	Baseline, 6 months, then annually
Injection volumes	∼0.2 ± 0.1 mL per half upper lip and lower lip
Primary outcome	Incidence of herpes labialis episodes (≥48 h, prodromal symptoms)
Baseline period	13 months pretreatment
Follow-up intervals	0-6, 6-18, 18-30, 30-42, and ≥42 months

The table summarizes the study design, patient population, treatment protocol, outcome definition, and statistical modeling strategy used for the longitudinal self-controlled analysis of recurrent herpes labialis episodes.

## RESULTS

### Study Population and Follow-Up

Between 2016 and 2025, a total of 141 patients with RHL underwent lip augmentation with HA filler. Following the application of predefined exclusion criteria during follow-up—including systemic antiviral therapy, antibiotic use for intercurrent infections, surgical procedures, or major medical conditions—the final analytical cohort comprised 90 patients. Of these, 83 (92.2%) were female and 7 (7.8%) male, with a mean age at baseline of 38.1 ± 9.8 years (range, 21-55 years). All included patients completed at least 2 treatment sessions and had longitudinal follow-up data available. Baseline recurrence frequency, assessed over the 13 months preceding the first filler session, ranged from 3 to 8 episodes per year, consistent with a population affected by RHL.

### Incidence of Herpes Labialis Episodes Across Treatment Intervals

**Figure 1.**
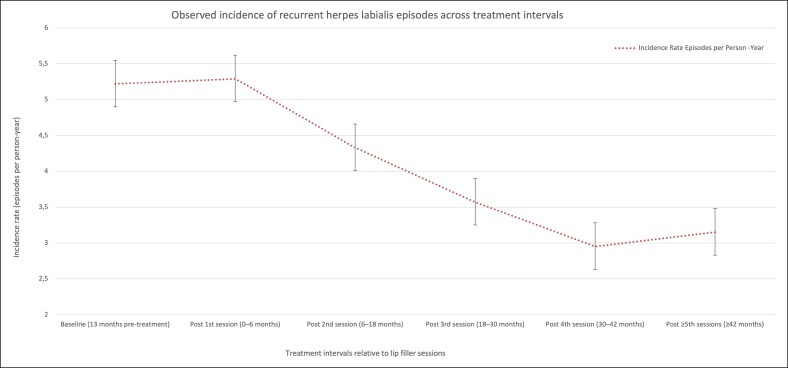
Observed incidence of recurrent herpes labialis episodes across treatment intervals. Observed incidence rates of recurrent herpes labialis episodes expressed as episodes per person-year across predefined baseline and posttreatment intervals relative to hyaluronic acid lip filler sessions. Error bars represent 95% CIs. Lines are shown for visual guidance only and do not imply continuity between intervals. Incidence rates are presented for descriptive purposes; statistical inference is based on generalized estimating equation models with a negative binomial distribution, as reported in Table 2.

Incidence rates of herpes labialis episodes were calculated as episodes per person-year for the baseline period and for each predefined posttreatment interval. At baseline, the overall incidence rate was 5.22 episodes per person-year. Following the first filler session, no appreciable change in recurrence frequency was observed. During the first 6 months after the initial treatment, the incidence rate remained comparable to baseline (5.29 episodes per person-year), with no statistically significant difference. In contrast, a progressive reduction in recurrence frequency emerged after subsequent treatment sessions. After the second session (6-18 months), the incidence rate decreased to 4.33 episodes per person-year. This reduction was greater after the third session (3.57 episodes per person-year) and after the fourth session (2.95 episodes per person-year). Beyond the fifth session, incidence rates remained relatively stable, ranging approximately between 3.0 and 3.3 episodes per person-year, suggesting a plateau effect. The temporal pattern of incidence rates across baseline and posttreatment intervals is illustrated in Figure 1.

### Association Between Treatment Exposure and Recurrence Rate

To formally evaluate the association between repeated HA filler sessions and herpes labialis recurrence, GEE models with a negative binomial distribution were fitted, accounting for within-patient correlation and varying follow-up duration. Compared with baseline, no significant change in recurrence risk was observed after the first session (IRR 1.01; 95% CI, 0.89-1.16; *P* = .855). Starting from the second session, a statistically significant reduction in recurrence risk was detected. Specifically, the IRR was 0.81 (95% CI, 0.72-0.91; *P* < .001) after the second session, 0.67 (95% CI, 0.57-0.78; *P* < .001) after the third session, and 0.55 (95% CI, 0.45-0.66; *P* < .001) after the fourth session. After 5 or more sessions, the IRR remained significantly lower than baseline (IRR 0.56; 95% CI, 0.46-0.68; *P* < .001), without evidence of further substantial reduction. Incidence rates and corresponding IRRs across treatment intervals are summarized in Table 2.

**Table 2.** Incidence Rates and IRRs of Recurrent Herpes Labialis Episodes Across Treatment Intervals

Interval	Incidence rate (episodes/person-year)	IRR vs baseline	95% CI	*P*-value
Baseline (13 months pretreatment)	5.22	Reference	—	—
Post-first session (0-6 months)	5.29	1.01	0.89-1.16	.855
Post-second session (6-18 months)	4.33	0.81	0.72-0.91	<.001
Post-third session (18-30 months)	3.57	0.67	0.57-0.78	<.001
Post-fourth session (30-42 months)	2.95	0.55	0.45-0.66	<.001
Post-≥fifth sessions (≥42 months)	3.15	0.56	0.46-0.68	<.001

Incidence rates are expressed as episodes per person-year. Incidence rate ratios (IRRs) and 95% CIs were estimated using generalized estimating equations with a negative binomial distribution, offset by person-time, and clustered by patient.

### Follow-Up and Cohort Retention

Among the 90 patients included in the final analytical cohort, not all individuals contributed data to every posttreatment interval, reflecting the longitudinal and real-world nature of follow-up. At the interval level, a total of 141 baseline observation periods were initially evaluable before the application of exclusion and censoring criteria, with decreasing numbers of observation intervals contributing data at later time points (138 after the second session, 127 after the third, 122 after the fourth, and 90 beyond the fifth session), as detailed in Supplemental Figure 1. The mean follow-up duration was 7.15 years (range, 1.33-11.04 years).

### Trend Analysis and Plateau Assessment

A stepwise trend analysis treating treatment exposure as an ordinal variable demonstrated a significant overall decline in recurrence rate with increasing number of sessions (IRR per session step 0.91; 95% CI, 0.88-0.94; *P* < .001). Piecewise modeling revealed that the reduction in recurrence risk was significant up to the fifth session, whereas no additional reduction was observed beyond this point, consistent with a stabilization or plateau effect.

### Sensitivity Analysis Using Post-First Session as Reference

To address potential recall bias associated with baseline recurrence estimates, an additional sensitivity analysis was performed using the post-first session interval as the reference category. Compared with the post-first session incidence rate, herpes labialis recurrence was significantly reduced after the second session (IRR 0.82; *P* < .001), with further reductions observed after the third (IRR 0.67; *P* < .001) and fourth sessions (IRR 0.56; *P* < .001). Recurrence rates after 5 or more sessions remained significantly lower than the post-first session level (IRR 0.57-0.62; *P* < .001), with no evidence of additional reduction, consistent with a plateau effect. These findings were concordant with the primary analysis, supporting the robustness of the observed association (Table 3).

**Table 3.** Sensitivity Analysis of Herpes Labialis Recurrence Using the Post-First Session as Reference

Follow-up interval	Incidence rate (episodes/person-year)	IRR vs post-first session	*P*-value
Post-first session (0-6 months)	5.29	Reference	—
Post-second session (6-18 months)	4.33	0.82	<.001
Post-third session (18-30 months)	3.57	0.67	<.001
Post-fourth session (30-42 months)	2.95	0.56	<.001
Post-≥fifth sessions (≥42 months)	3.02-3.27	0.57-0.62	<.001

Sensitivity analysis comparing herpes labialis recurrence rates across treatment intervals using the post-first session as the reference category. Incidence rate ratios (IRRs) were estimated using generalized estimating equations with a negative binomial distribution, accounting for within-patient correlation and variable follow-up duration through an offset for person-time at risk.

### Summary of Main Findings

Overall, these results indicate that repeated lip augmentation with HA filler was associated with a statistically significant, progressive reduction in the incidence of RHL episodes, becoming evident after the second treatment session and reaching a stable plateau after multiple sessions. No effect was observed after a single treatment session.

## DISCUSSION

In this longitudinal self-controlled cohort, repeated lip augmentation with HA filler was associated with a progressive and statistically significant reduction in the incidence of RHL episodes, becoming evident after the second treatment session and stabilizing after multiple sessions. The absence of any measurable change after the first filler session does not support immediate placebo effects or a simple regression-to-the-mean explanation.^12^ Instead, the progressive reduction observed only after repeated exposures is consistent with a cumulative or threshold-dependent process rather than a nonspecific time-related phenomenon. From a clinical perspective, a reduction from ∼5 to 3 herpes labialis episodes per year may represent a meaningful improvement for affected patients by reducing symptomatic burden and episodic treatment needs. However, no minimal clinically important difference was predefined, and patient-reported quality-of-life outcomes were not formally assessed. These interpretations remain speculative and cannot be confirmed within the observational design of the present study. Such a pattern is more consistent with an exposure-related modification of local conditions influencing viral reactivation.

Several nonmutually exclusive hypotheses may plausibly account for the observed association. HA filler may induce long-lasting mechanical and structural changes in lip tissue, reducing microtrauma and inflammatory stimuli associated with HSV reactivation, and may also interact with cellular receptors involved in immune modulation and tissue homeostasis, potentially contributing to a local microenvironment less permissive to viral reactivation.^13^ Improved barrier function and hydration of the vermilion may reduce exposure to environmental triggers, including ultraviolet radiation and cold-induced irritation. Importantly, the delayed onset of the effect argues against an acute antiviral action and favors mechanisms requiring repeated or sustained tissue modification. No direct mechanistic measurements were performed, and these hypotheses are proposed solely to contextualize the observed association. All proposed mechanisms should be regarded as speculative and are presented solely to support biological plausibility rather than to imply a causal relationship. The observed temporal pattern is more consistent with cumulative tissue-level changes across repeated treatments rather than with acute postprocedural effects.

To the best of our knowledge, this is the first longitudinal study systematically quantifying changes in herpes labialis recurrence following aesthetic lip augmentation. Previous literature on herpes labialis has primarily focused on antiviral prophylaxis, immunomodulatory approaches, or trigger avoidance strategies, with limited exploration of structural or tissue-based interventions.^14^ In contrast to the present findings, existing literature addressing herpes labialis in the context of aesthetic procedures has primarily described HSV reactivation as a rare but recognized complication of perioral interventions. Published evidence in this area consists largely of isolated case reports, small case series, and expert consensus statements documenting acute HSV reactivation following HA dermal filler injections. These reports have focused on short-term postprocedural outcomes and risk mitigation strategies, such as antiviral prophylaxis in selected patients but have not systematically examined longitudinal changes in recurrence patterns over repeated treatments.^8,15,16^ The focus of the present study was not to assess acute postprocedural herpes reactivation but to evaluate longitudinal changes in recurrence frequency in a selected population of patients with clinically significant RHL. In this context, the observed association between repeated lip augmentation and reduced recurrence rates does not contradict existing literature describing acute reactivation as a procedural complication but rather addresses a distinct clinical question that has not been previously examined. Both phenomena may coexist, because they address different temporal and clinical dimensions of HSV reactivation.

Self-controlled designs similar to the one adopted here have been widely used in other medical fields to study recurrent events when randomized controls are impractical, including vaccine safety studies and pharmacovigilance analyses.^9^ The present findings extend this methodological approach to an aesthetic-dermatological setting and suggest that interventions not primarily intended for infectious disease management may nonetheless influence recurrence patterns.

Several methodological strengths deserve consideration. First, the self-controlled longitudinal design inherently controls for time-invariant individual confounders, such as genetic susceptibility, baseline immune competence, and lifetime exposure history. Second, the use of models specifically designed for recurrent events, with appropriate offsets for person-time and clustering by patient, ensures that the statistical analysis aligns with the structure of the data. Third, the long observation period allowed the identification of progressive effects that would not be detectable in shorter studies. Finally, the absence of systemic antiviral therapy throughout follow-up reduces a major source of confounding.

From a clinical perspective, the findings suggest that repeated lip augmentation with HA filler may be associated with a sustained reduction in herpes labialis recurrence in selected patients. This observation does not support the use of filler as a treatment for herpes labialis, but it may inform patient counseling and risk-benefit discussions in individuals with a history of frequent recurrences. From a research standpoint, the results provide a rationale for prospective studies, ideally with controlled designs, to further explore this association and clarify underlying mechanisms. Future investigations could incorporate virological confirmation and experimental approaches to further characterize these mechanisms.

### Limitations

The observational nature of the study precludes definitive causal inference. Although the temporal pattern and internal consistency of the findings argue against simple time-related explanations, unmeasured time-varying confounders cannot be fully excluded, including changes in lifestyle, stress exposure, or environmental triggers over time. The study was not prospectively registered and no prespecified protocol was available, because the analysis was initiated in response to an unexpected clinical observation; this limits the ability to fully exclude analytical flexibility or selective reporting and should be considered when interpreting the findings. Accordingly, the baseline interval should be interpreted as an operational reference period rather than a definitive estimate of long-term recurrence frequency. Episode identification relied primarily on patient report, which introduces the possibility of recall bias and reporting variability. However, the self-controlled longitudinal design mitigates this limitation, because each participant served as their own control across all observation intervals, reducing the impact of stable individual reporting tendencies over time. In addition, episodes were diagnosed on clinical grounds without routine virological confirmation, which may allow for outcome misclassification; however, within a self-controlled longitudinal design, any nondifferential misclassification would be expected to bias estimates toward the null rather than generate a progressive, session-dependent association. Although topical acyclovir use was permitted, adherence and timing were not objectively assessed, and changes in treatment behavior over time could theoretically influence episode reporting. Importantly, topical antivirals primarily affect episode duration and symptom severity rather than the incidence of reactivation events, which was the primary outcome of this study and was expressed as episodes per person-year. Differential dropout may have introduced survivor bias, as patients who continued returning for multiple treatment sessions may differ systematically from those who discontinued earlier. Although all available person-time was included and variable follow-up was accounted for analytically, this limitation should be considered when interpreting longitudinal trends.

The lack of a parallel untreated control group may be viewed as a limitation, although such a control is difficult to implement in the context of elective aesthetic treatments. The chosen self-controlled approach represents a methodologically appropriate alternative under these constraints. Finally, the study population was derived from a single clinical setting, which may limit generalizability.

## CONCLUSIONS

In this longitudinal self-controlled study, repeated HA lip filler treatments were associated with a progressive reduction in RHL incidence after multiple sessions. Although causality cannot be definitively established, the consistency, temporal structure, and statistical robustness of the findings support a treatment-associated effect and merit further investigation in future studies. These findings should not be interpreted as evidence supporting HA filler as a therapeutic intervention for herpes labialis.

## Supplementary Material

ojag060_Supplementary_Data
