# Supplementary material for: The Association Between Repeated Lip Augmentation With Hyaluronic Acid Filler and Recurrence of Herpes Labialis: A Longitudinal Self-controlled Study
Source: Aesthet Surg J Open Forum. 2026 Apr 1;8:ojag060. doi: 10.1093/asjof/ojag060 (PMC13151027; doi:10.1093/asjof/ojag060)
Supplement: ojag060_Supplementary_Data [file ojag060_supplementary_data.zip › Supplemental Figure Legend.docx]

**Supplemental Figure Legend**

**Supplemental Figure 1.** Flow diagram of patient selection, exclusions, follow-up, and final analytical cohort. Flow diagram illustrating patient identification, eligibility assessment, application of exclusion criteria, censoring during follow-up, and inclusion in the final longitudinal self-controlled analysis. Reasons for exclusion and censoring are detailed, including baseline ineligibility, intercurrent exclusion criteria during follow-up, treatment discontinuation, and end of observation period.
