# Supplementary material for: The Association Between Repeated Lip Augmentation With Hyaluronic Acid Filler and Recurrence of Herpes Labialis: A Longitudinal Self-controlled Study
Source: Aesthet Surg J Open Forum. 2026 Apr 1;8:ojag060. doi: 10.1093/asjof/ojag060 (PMC13151027; doi:10.1093/asjof/ojag060)
Supplement: ojag060_Supplementary_Data [file ojag060_supplementary_data.zip › Appendix.docx]

# Appendix. Standardized Data Collection Checklist Used For the Longitudinal Assessment of Recurrent Herpes Labialis

## Section A – Patient Eligibility

- History of recurrent herpes labialis (≥3 episodes/year): Yes / No
- Age ≥18 years: Yes / No
- Systemic antiviral therapy at baseline or during follow-up: Yes / No (exclusion if Yes)
- Immunocompromising conditions (e.g., autoimmune disease, immunosuppressive therapy): Yes / No (specify)
- Major systemic illness during follow-up: Yes / No (specify)

## Section B – Baseline Assessment (Pre-treatment)

- Duration of herpes labialis history (years): ____
- Number of episodes in the 13 months prior to first filler session: ____
- Typical triggers (check all that apply): Stress / UV exposure / Fever / Hormonal / Cold exposure/ Other
- Usual management prior to study (topical antivirals only): Yes / No

## Section C – Treatment Exposure

- Date of filler session: ____
- Session number: ____
- Injection technique (vertical standardized): Yes / No
- Total hyaluronic acid volume injected (mL): ____
- Adverse events related to procedure: None / Yes (specify)

## Section D – Herpes Episode Recording (Follow-up)

- Date of episode onset: ____
- Date of episode resolution: ____
- Presence of prodromal symptoms: Yes / No
- Duration of episode (days): ____
- Use of topical antiviral therapy: Yes / No
- Corroborating documentation available (e.g., photograph) / clinical examination: Yes / No

## Section E – Time-varying Confounders

- Intercurrent infections since last visit: Yes / No (specify)
- Systemic antibiotic use: Yes / No (specify)
- Surgical procedures since last visit: Yes / No (specify)
- Major life stressors reported: Yes / No (specify)

This standardized checklist was used for systematic data collection during routine clinical follow-up; baseline data were reconstructed retrospectively, as detailed in the main manuscript.
