# Supplementary figures and images for: The Association Between Repeated Lip Augmentation With Hyaluronic Acid Filler and Recurrence of Herpes Labialis: A Longitudinal Self-controlled Study
Source: Aesthet Surg J Open Forum. 2026 Apr 1;8:ojag060. doi: 10.1093/asjof/ojag060 (PMC13151027; doi:10.1093/asjof/ojag060)

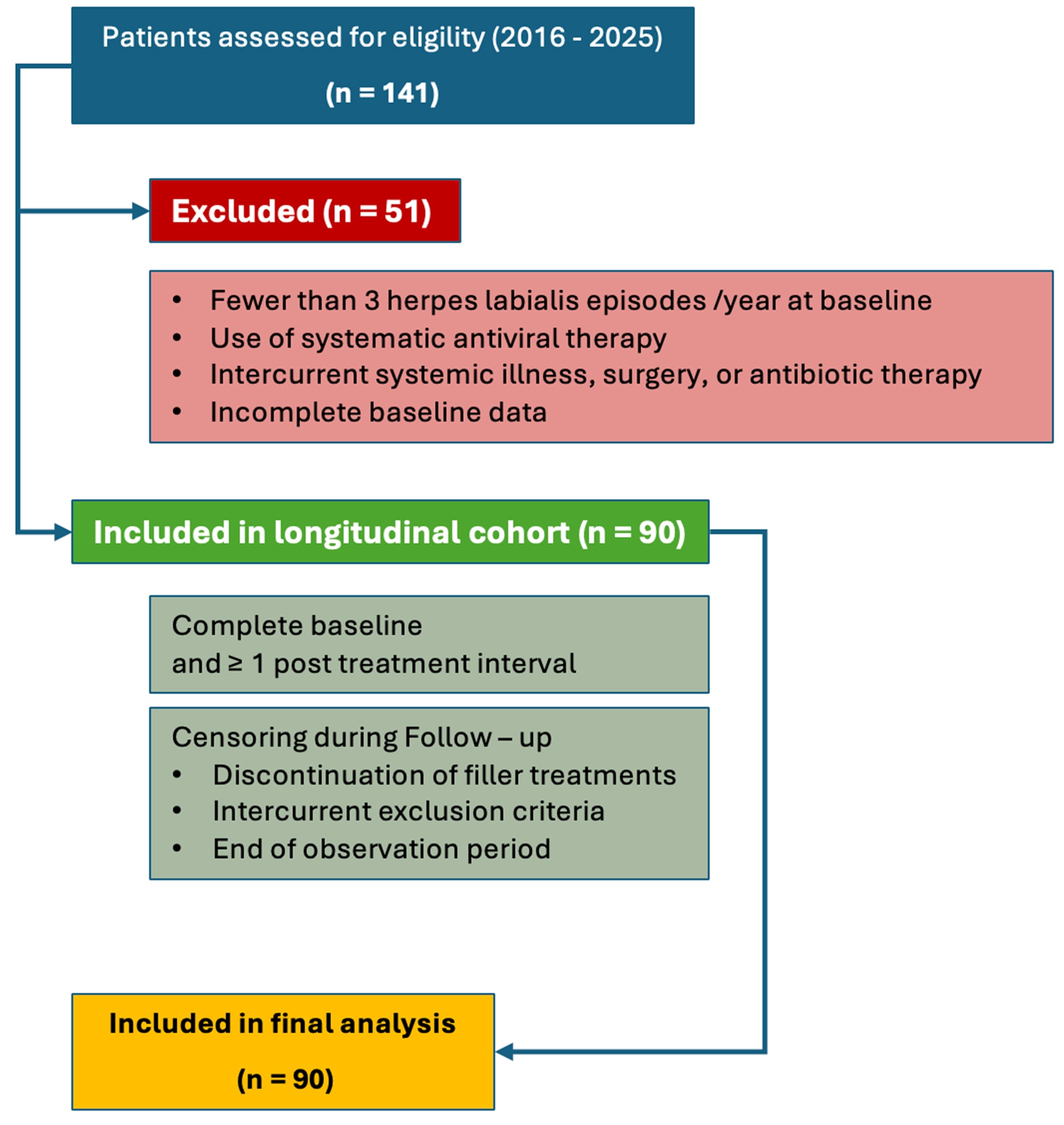

Supplement: ojag060_Supplementary_Data [file ojag060_supplementary_data.zip › Supplemental_Figure_1.jpg]

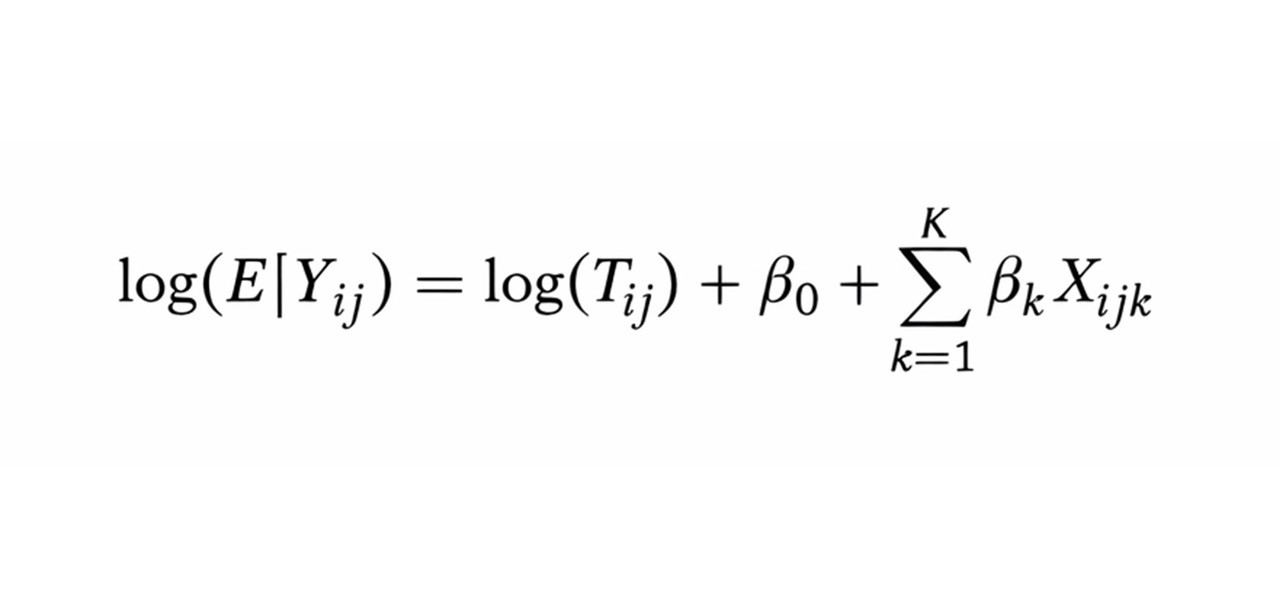

Supplement: ojag060_Supplementary_Data [file ojag060_supplementary_data.zip › Supplemental_Figure_2.jpg]
